# Immune checkpoint blockade in ovarian cancer

**DOI:** 10.1007/s12254-016-0267-3

**Published:** 2016-06-02

**Authors:** Lukas Weiss, Florian Huemer, Brigitte Mlineritsch, Richard Greil

**Affiliations:** 3rd Medical Department of Hematology, Medical Oncology, Hemostaseology, Rheumatology and Infectious Disease, Salzburg Cancer Research Institute (SCRI), Muellner Hauptstraße 48, 5020 Salzburg, Austria

**Keywords:** Ovarian cancer, Immunotherapy, Immune checkpoint, PD-1, PD-L1, Ovarialkarzinom, Immuntherapie, Immune Checkpoint, PD-1, PD-L1

## Abstract

Increased numbers of tumour infiltrating T‑cells have long been associated with a better prognosis in ovarian cancer, which has led to the general assumption of a relevant impact of T‑cellular anti-tumour immunity in this disease. As a consequence of this knowledge, a multitude of immunologic therapies has emerged over the past years. Although some reports could evidence a successful induction of anti-tumour T‑cells, in general, these attempts did not translate into clinically significant activity. As has already been shown in other tumour entities, immune checkpoint blockade – mainly antibodies directed against PD-1 and PD-L1 – could possibly become a real “game changer” in ovarian cancer in the future.

## Background

The prognostic significance of tumour infiltrating T‑cells (TILs) in the setting of advanced ovarian cancer (stages III and IV) was already described as early as 2003 [1]. Zhang and colleagues analysed 174 patients and evidenced that the presence of TILs was associated with a significantly longer overall survival (OS) with a 5-year OS of 38 % in contrast to only 4.5 % in the cohort without TILs. These data have been corroborated in several further studies and have been summarized in a meta-analysis including 1815 patients [2]. By further characterizing TILs, the positive prognostic impact could be attributed to the subgroup of CD8 positive intratumoural T‑cells. Therefore it can be hypothesized that the increased presence of TILs is caused by immunologic recognition of aberrant tumour cells, which ultimately results in improved immunologic tumour control.

Regulatory T‑cells are important mediators of peripheral immune tolerance and are able to suppress T‑cell responses at multiple levels. Regulatory T‑cells can also suppress T‑cell mediated anti-tumour responses against ovarian cancer, being one of the first tumour entities in which the role of regulatory T‑cells was described. Curiel et al. reported that an increased presence of intratumoural regulatory T‑cells was associated with significantly shorter overall survival in 70 patients with ovarian cancer [3]. This may be explained by an effective suppression of the anti-tumour responses exerted by CD8 positive TILs, which in turn leads to the observed worse clinical outcome. These findings add to the body of evidence supporting the central role of T‑cells in anti-tumour immunity in ovarian cancer.

## Immune checkpoint inhibitors – mode of action

Immune checkpoint-inhibitors are often thought to represent a paradigm shift in cancer therapy. In stark contrast to most other forms of cancer therapy the cancer cell itself does not constitute the primary target, but immune cells or immune interactions do. As opposed to previous immunotherapeutic approaches, immune checkpoint-inhibitors are rather aimed at unleashing a pre-existing anti-tumour response than at a general activation of the immune system. Tumours may develop different strategies to evade an immunologic attack by hijacking physiologic mechanisms intended to limit immune responses, the so-called adaptive immune resistance. There is a multitude of so-called immune checkpoints, which regulate cellular interactions between T‑cells and antigen presenting cells, cells of the innate immune system (such as tissue macrophages), as well as tumour cells [4]. So far the greatest attention has been drawn to the molecules cytotoxic T‑lymphocyte-associated protein 4 (CTLA-4), programmed cell death 1 (PD-1) and programmed-death ligand 1 (PD-L1).

CTLA-4 is expressed on activated T‑cells and ligation inhibits further T‑cell activation. Antibodies directed against CTLA-4 (e. g., ipilimumab or tremelimumab) can maintain already activated T‑cells by blocking inhibitory signalling through CTLA-4. Ipilimumab is approved by the European Medicines Agency for the treatment of non-resectable or metastatic melanoma. The molecule PD-1 and its ligand PD-L1 play an important role in the interaction between tumour-specific T‑cells and tumour cells. T‑cell activation and cytotoxic effector functions are inhibited by ligation of PD-1 on the T‑cell by PD-L1 on the tumour cell. Both antibodies against PD-1 or PD-L1 can be used for blocking this signal and may thereby unleash an active anti-tumour response. The anti PD-1 antibodies nivolumab and pembrolizumab have been approved by the European Medicines Agency for the treatment of non-resectable or metastatic melanoma. Nivolumab is also approved for the second line treatment of metastatic squamous non-small cell lung cancer. Several other immune checkpoint-inhibitors are currently being developed and tested for clinical efficacy in nearly all tumour entities.

## Immune checkpoint inhibitors – clinical activity

Besides their distinctive features regarding their mode of action, foremost the clinical efficacy of immune checkpoint inhibitors has attracted great interest by the medical and scientific community as well as the general public. In a pooled analysis of 4846 patients with metastatic melanoma, treatment with the anti-CTLA-4 antibody Ipilimumab resulted in long-term tumour control in roughly 20 % of patients [5]. Of note is that the majority of these patients has been treated with the approved regimen of four doses of ipilimumab given at 3‑week intervals, which nevertheless resulted in effective immunologic tumour-control of up to 10 years in a subgroup of patients suffering from a metastatic cancer. Interestingly, disease control was observed regardless of remission status – another distinguishing feature in comparison to other established cancer therapies. As a result, most clinical trials using immune checkpoint-inhibitors report the so called “disease-control-rate”, which unifies the response categories of stable disease, and partial and complete response.

## Immune checkpoint inhibitors in ovarian cancer

Expression of PD-L1 on tumour cells is considered to represent a major immune evasion strategy in cancer. In ovarian cancer, patients with higher tumoural expression-levels of PD-L1 exhibited significantly shorter overall survival when compared to patients with lower expression levels [6]. First data supporting immune checkpoint inhibitors as a potentially valuable therapeutic approach in ovarian cancer were observed in larger Phase-1 trials of the anti PD-1 antibody nivolumab and the anti PD-L1 antibody BMS-93655, including in patients with various solid tumours. Two trials specifically aimed at ovarian cancer using the anti PD-L1 antibody avelumab and the anti PD-1 antibody pembrolizumab were presented at the annual meeting of the American Society of Clinical Oncology in 2015 and corroborate the previously observed results (Tab. 1):

**Tab. 1:** PD-1 & PD-L1 blockade in ovarian cancer

Substance	Target	*N*	CR	PR	SD	Disease control rate
Nivolumab [9]	PD-1	20	2	1	6	9/20 (45 %)
BMS-936559 [10]	PD-L1	17	0	1	3	4/17 (23 %)
Avelumab [7]	PD-L1	75	0	8	33	41/75 (54.7 %)
Pembrolizumab [8]	PD-1	26	1	2	6	9/26 (34.6 %)

In a Phase-1b expansion study 75 patients were treated with avelumab [7]. Although none of the patients experienced a complete response, a partial response was seen in 8 patients, whereas 33 patients were classified as having a stable disease, leading to a disease control rate of 54.7 %. More than two-thirds of the patients received three or more lines of therapy prior to avelumab treatment. Therefore, avelumab was deemed clinically active in this heavily pre-treated cohort.

In a Phase-1b study (KEYNOTE-028) 26 patients were treated with pembrolizumab [8]. In contrast to the avelumab trial, a PD-L1 expression level of ≥1 % of tumour cells was required for study entry. One complete response, two partial responses and six patients with stable disease correspond to a disease control rate of 34.6 %. Again, the duration of response was independent of response according to Response Evaluation Criteria in Solid Tumors (RECIST) criteria. In this study, the fraction of heavily pre-treated patients was even higher with 88.5 % of patients having received three or more previous lines of therapy.

As known from trials for metastatic melanoma and lung cancer, anti PD-1 antibody as well as anti PD-L1 antibody treatment showed a beneficial toxicity profile. Given an overall adverse event rate of 70 %, only 8.0 % (avelumab trial) or 3.8 % (pembrolizumab trial) of patients experienced grade 3/4 toxicities. In this regard, we underscore the importance of the correct management of immune-related adverse events, which dramatically differ from chemotherapy associated adverse events. Although generally less frequent and less severe, these immune-related adverse events may require rapid therapeutic intervention such as systemic corticoids, which can effectively prevent possibly life-threatening complications.

## Outlook

In summary, existing data on immune checkpoint inhibitors in ovarian cancer so far is highly promising and further proof is eagerly awaited in order to implement this novel therapeutic approach in the clinics. Determining who will benefit and who will not remains a crucial problem that must be solved. Different genetic stability might be a possible starting point; the more instable a tumour becomes, the higher the probability of the tumour expressing aberrant proteins, which can be recognized by the immune system. Therefore, the so-called BRCAness could represent a possible predictive marker for response to immune checkpoint inhibitors in ovarian cancer.
